# Ultrahigh‐Throughput Screening of an Artificial Metalloenzyme using Double Emulsions**

**DOI:** 10.1002/anie.202207328

**Published:** 2022-10-27

**Authors:** Jaicy Vallapurackal, Ariane Stucki, Alexandria Deliz Liang, Juliane Klehr, Petra S. Dittrich, Thomas R. Ward

**Affiliations:** ^1^ Department of Chemistry University of Basel Mattenstrasse 24a 4058 Basel Switzerland; ^2^ Department of Biosystems Science and Engineering ETH Zurich Mattenstrasse 26 4058 Basel Switzerland; ^3^ National Competence Center in Research (NCCR) Molecular Systems Engineering Basel Switzerland

**Keywords:** Allylic Deallylation, Artificial Metalloenzymes, Directed Evolution, Droplet Microfluidics, High-Throughput Screening

## Abstract

The potential for ultrahigh‐throughput compartmentalization renders droplet microfluidics an attractive tool for the directed evolution of enzymes. Importantly, it ensures maintenance of the phenotype‐genotype linkage, enabling reliable identification of improved mutants. Herein, we report an approach for ultrahigh‐throughput screening of an artificial metalloenzyme in double emulsion droplets (DEs) using commercially available fluorescence‐activated cell sorters (FACS). This protocol was validated by screening a 400 double‐mutant streptavidin library for ruthenium‐catalyzed deallylation of an alloc‐protected aminocoumarin. The most active variants, identified by next‐generation sequencing, were in good agreement with hits obtained using a 96‐well plate procedure. These findings pave the way for the systematic implementation of FACS for the directed evolution of (artificial) enzymes and will significantly expand the accessibility of ultrahigh‐throughput DE screening protocols.

## Graphical abstract

An artificial metalloenzyme was screened in vivo using a high‐throughput microfluidics‐based assay. Single *E. coli* cells expressing streptavidin were encapsulated in double emulsion droplets together with a fluorogenic substrate and a cofactor catalyzing the reaction to yield a fluorescent product. Using this method, a 400‐member library was screened, and the same hits found previously in a 96‐well plate assay were identified.

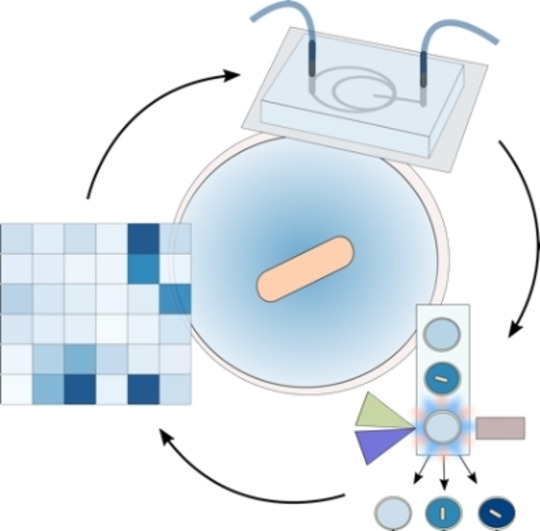

## Introduction

Progress in protein engineering, DNA sequencing, and bioinformatics has enabled the engineering of enzymes to a specific need. While enzyme properties have evolved over thousands of generations by natural selection, directed evolution offers tools to accelerate and streamline this process by screening enzyme libraries and selecting the most promising candidates for a given function. Early directed evolution campaigns have yielded enzymes with increased solvent tolerance^[1]^ or significantly enhanced catalytic activity^[2]^ and selectivity.[^3^, ^4^, ^5^] Despite these achievements, the full potential of directed evolution has not yet been realized due to the requirement that the genotype‐phenotype linkage be preserved during the entire screening process. Accordingly, most directed evolution campaigns rely on screening libraries in microtiter plates (MTPs). Such screening campaigns are time‐ and resource‐intensive and scale exponentially with the number of amino acid positions screened simultaneously.[^6^, ^7^] These aspects impose severe limitations on the library size, thus limiting scientists either to the screening of single positions iteratively or only a limited portion of the possible variant landscape.

Within the realm of evolvable enzymes, artificial metalloenzymes (ArMs) have recently attracted increasing attention. Consisting of an abiotic metal cofactor anchored within an evolvable protein scaffold, these hybrid catalysts can exhibit unique new‐to‐nature reactivities.^[8]^ In this context, ArMs based on the biotin‐streptavidin (biot‐Sav) technology have been genetically engineered to catalyze numerous reactions including metathesis^[9]^ as well as transfer hydrogenation, hydroamination, and hydroxylation.^[10]^ Other versatile ArM‐scaffolds include carbonic anhydrase,^[11]^ hemoproteins,[^12^, ^13^, ^14^] prolyl oligopeptidase,^[15]^ four‐helix bundles,^[16]^ the lactococcal multiresistance regulator,^[17]^ or even de novo designed metallopeptides.^[18]^ Building on the pioneering work of Meggers and co‐workers,[^19^, ^20^, ^21^] streptavidin‐based ArMs that catalyze the deallylation of allyl‐carbamate‐protected coumarin were previously screened in 96‐well plates.[^22^, ^23^] An artificial deallylase (ADAse) contained in the periplasm of *E. coli* cells was optimized by simultaneously randomizing two amino acid residues, to afford a 20‐fold improvement in activity in a single round.^[23]^ These results highlighted that (i) ArMs can be evolved to catalyze new‐to‐nature and bioorthogonal reactions, (ii) the periplasm of an *E. coli* offers a hospitable environment to compartmentalize ArMs while maintaining the phenotype‐genotype linkage and (iii) in contrast to iterative saturation mutagenesis, the systematic screening of multiple positions simultaneously enables the identification of potential synergistic mutations. However, the lack of high‐throughput screening tools typically restricts these studies to the MTP screening of libraries containing less than 500 mutants.

Advances in microfluidics, and particularly in droplet‐based microfluidics, over the past 20 years have led to the development of tools enabling high‐throughput screening of large libraries of enzymes.[^24^, ^25^] Such tools are based on the encapsulation of single genetic variants (single cells or DNA molecules) in aqueous compartments (droplets) together with the reaction components. Monodisperse droplets can be produced on‐chip at throughputs of several thousand hertz, resulting in the encapsulation of large libraries within seconds to minutes. Each droplet, isolated from its surroundings by oil, provides a means of maintaining the phenotype‐genotype linkage. Additionally, the use of fluorogenic reactions has enabled further advances in high‐throughput sorting—such as on‐chip fluorescence‐activated droplet sorting—and has led to the optimization of various enzymes.[^24^, ^25^, ^26^, ^27^, ^28^, ^29^, ^30^] However, even though recent publications have reported on high‐throughput droplet sorting,^[26]^ the droplet sorting speed of most microfluidic platforms is typically significantly lower (≈a few Hz)^[31]^ than the achievable production rate (≈several kHz),^[32]^ and decreases even further if the sample is sorted into more than two distinct populations. Moreover, on‐chip sorting of single emulsion water‐in‐oil droplets requires custom‐engineered chips and software, thus limiting the use of microfluidic screening to highly specialized teams.

**Figure 1:**
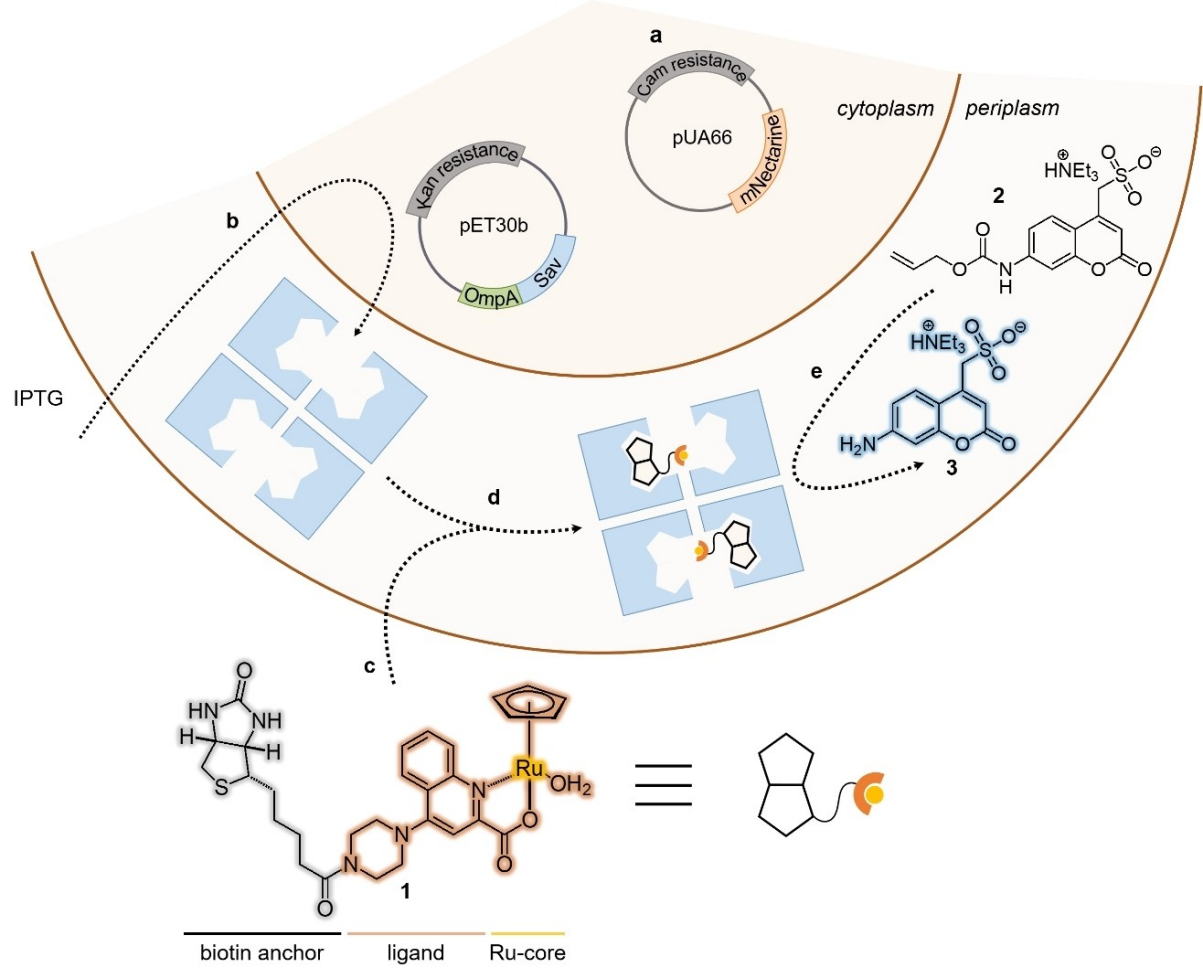
Model system used for the directed evolution of artificial metalloenzymes using a microfluidics‐based screening assay. a) mNectarine is encoded on a pUA66 vector and is constitutively expressed and compartmentalized in the cytoplasm, enabling the fluorescence detection of DE droplets containing an *E. coli* cell. b) Sav is encoded on a pET30b vector and induction with IPTG leads to the overexpression and secretion of Sav into the periplasm of *E. coli*, where it forms a homotetrameric protein. c) The biotinylated ruthenium cofactor **1** passively diffuses through the outer‐membrane into the periplasm and d) is anchored in the binding pocket of Sav, affording the artificial deallylase (ADAse). e) Uncaging of the allyl‐carbamate protected substrate **2** yields the fluorescent aminocoumarin **3**, which can be detected and sorted by FACS.

The challenges associated with screening of single emulsion droplets may be addressed through the use of double emulsion (water‐in‐oil‐in‐water) droplets (DEs), which, as a result of their aqueous exterior, are compatible with commercially available ultra‐fast flow cytometers and FACS available at many academic facilities.[^33^, ^34^, ^35^] The production of DEs for directed evolution has typically involved the use of batch methods, which lead to polydisperse droplets with multiple inner aqueous phase compartments.[^36^, ^37^, ^38^] Recent developments, however, have led to the high‐throughput on‐chip generation of water‐in‐oil‐in‐water droplets, analogous to single emulsion droplets, and their use in the directed evolution of natural enzymes.[^39^, ^40^] The use of DEs enables compartmentalization of single genetic variants (i.e., genotype) with the corresponding reaction products (i.e., phenotype), and high‐throughput sorting of the DEs into several populations can be achieved using FACS. Importantly, the sorting throughput is comparable to the droplet production rate (kHz), thus enabling streamlining and automatization of the entire process. Furthermore, the use of widely available FACS instrumentation renders droplet sorting more accessible to a broader scientific community. Accordingly, this strategy is an attractive tool for high‐throughput directed evolution of enzymes. Here, we describe an ultrahigh‐throughput assay based on DE microfluidics for the in vivo directed evolution of an artificial deallylase (ADAse) based on biot‐Sav technology (Figure 1). The method is validated initially by carrying out a model enrichment and further validated by screening a 400 double‐mutant library.^[23]^ The rapid screening enabled by this protocol has the potential to transform directed evolution of enzymes. Indeed, while one researcher screening sixteen 96‐well plates per week would require approximately six years to screen 500 000 variants, the DE method shortens this workflow to a single week, more than a 300‐fold reduction in turnaround time.

## Results and Discussion

The catalytic system used in this work is a previously described ArM^[22]^ based on Sav compartmentalized in the periplasm of *E. coli* and the biotinylated ruthenium cofactor **1** (Figure 1).^[23]^ The *E. coli* are equipped with two different plasmids. The first is a pUA66 vector encoding mNectarine with chloramphenicol (cam) resistance (Figure 1a). The red‐fluorescent protein mNectarine is therefore expressed constitutively. The second plasmid is a pET30b vector encoding a T7‐tagged Sav fused to the signal peptide of the outer membrane protein A (OmpA) with kanamycin (kan) resistance. Upon addition of isopropyl β‐D‐1‐thiogalactopyranoside (IPTG), Sav expression is induced in the cytoplasm, and the protein is subsequently secreted to the periplasm, where it forms the homotetrameric protein consisting of four β‐barrels that can bind up to four equivalents of biotin^[12]^ (Figure 1b). Following expression, the cofactor **1** is added and the ADAse self‐assembles in the periplasm as a result of the high affinity of Sav for biotin (Figure 1c, d). The allylcarbamate‐protected aminocoumarin 2 is deprotected by the catalyst to afford the fluorescent aminocoumarin 3, which can be used to evaluate the catalytic activity of the encapsulated ADAse (Figure 1e).

**Figure 2:**
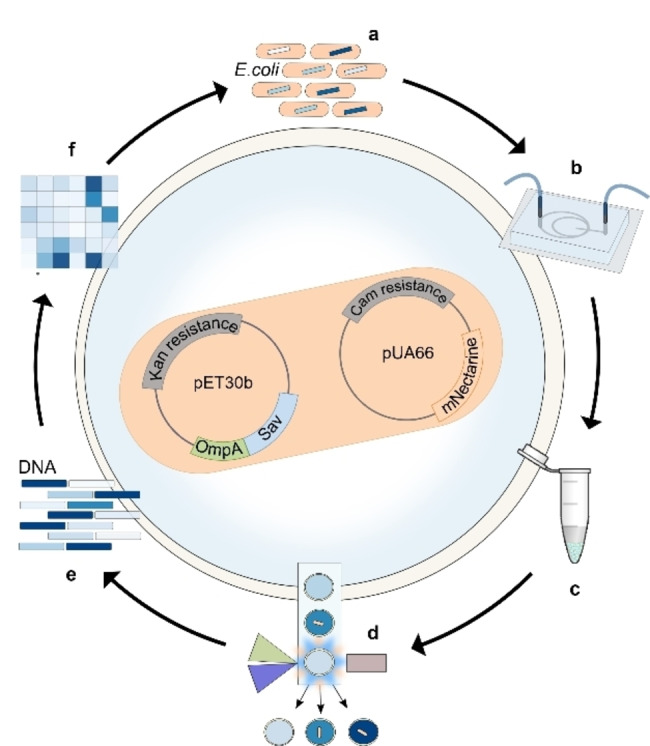
Assay workflow for the screening of ArMs in double emulsion droplets. a) A library of *E. coli* harboring Sav in their periplasm and mNectarine is produced and incubated with cofactor **1** in phosphate‐buffered saline. b) The *E. coli* library and the substrate **2** are fed onto a microfluidic chip in two separate solutions and encapsulated together in double emulsion droplets (DEs). c) After incubation at 37 °C off‐chip, d) the DEs are subjected to FACS, which enables a dual‐channel sorting to enrich a population of droplets containing an *E. coli* cell (as highlighted by the FI of mNectarine, *λ*_ex_=560 nm, *λ*_em_=580 nm) and displaying high catalytic activity (as revealed by the FI of aminocoumarin **3**, *λ*_ex_=405 nm, *λ*_em_=460 nm). e) Plasmid extraction of the sorted droplets yields plasmid DNA, which is amplified by PCR to enable f) NGS analysis of the sorted library. Hits can be transformed back into *E. coli* to reiterate the cycle.

For the ultrahigh‐throughput screening of the ADAse, we utilized the approach presented in Figure 2. In brief, mNectarine‐labeled *E. coli* cells expressing Sav were incubated with the ruthenium cofactor **1** and then encapsulated with an allyl‐carbamate protected aminocoumarin substrate **2** (Figure 2b) in DEs on a microfluidic chip. The resulting droplets were collected off‐chip, incubated under the desired reaction conditions, and then sorted using FACS based on the fluorescence intensity (FI) of both mNectarine and coumarin (Figure 2c, d). The presence of an mNectarine signal enabled the sorting of DEs containing an *E. coli* cell, while the coumarin fluorescence served as a readout for catalytic activity. After sorting, the DEs were ruptured, the plasmid was extracted, and the gene of interest was PCR‐amplified and analyzed by next generation sequencing (NGS) (Figure 2e, f). Note, the hits identified in this way may be subjected to another round of the assay to iteratively evolve the enzyme of interest.

### Characterization of the DE‐Based Screening Assay

**Figure 3:**
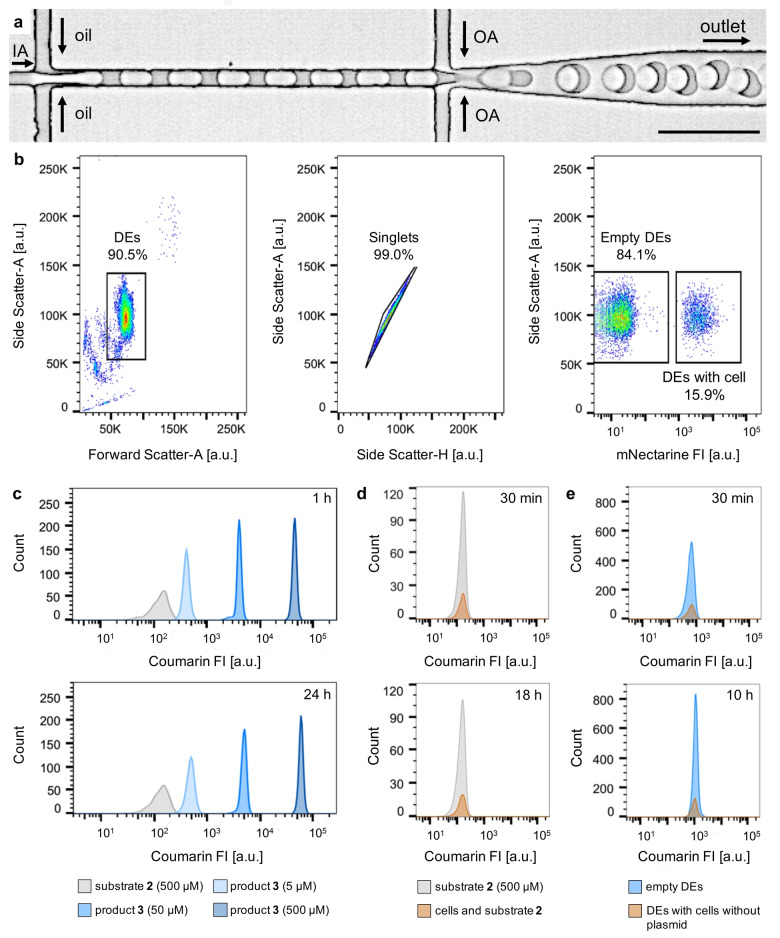
DE formation and reaction monitoring by FACS. a) Micrograph of DE formation. Monodisperse DEs co‐encapsulating *E. coli* cells, cofactor **1** and substrate **2** are produced using a PDMS‐based microfluidic device. IA=inner aqueous phase, OA=outer aqueous phase, oil=oil phase, scale bar: 50 μm. b) Left: Flow cytometer light scatter gate of a DE sample displaying 10 000 events randomly sampled, revealing the size and shape distribution of the sample. In this case ≈91 % of the sampled DEs are monodisperse. Middle: Flow cytometer light scatter gates of homogeneous DEs from the selected DE subpopulation. This plot was used to distinguish DEs with single aqueous cores from DEs with multiple aqueous cores. Right: mNectarine FI of the selected DE subpopulation enabling the sorting of DEs containing an *E. coli* cell and empty DEs. c) Coumarin FI distribution of four different DE samples, encapsulating respectively 500 μM substrate **2** or different product **3** concentrations (5, 50 and 500 μM), analyzed 1 h (top) and 24 h (bottom) after encapsulation. d) Coumarin FI distribution of DEs encapsulating 500 μM substrate **2** and *E. coli* featuring wt‐Sav, analyzed 30 min and 18 h after encapsulation, highlighting that substrate **2** is inert in the absence of the cofactor **1**. e) Coumarin FI distribution of DEs encapsulating substrate **2** (500 μM) and cells without plasmid for Sav expression (pre‐incubated with 5 μM of cofactor **1**), obtained 30 min and 10 h after encapsulation.

Monodisperse DEs with a diameter of about 15 μm were produced on polydimethylsiloxane (PDMS) chips at rates >6000 Hz (Supporting Information Figure 1 and Figure 3a).^[35]^ With on‐chip production, monodisperse droplets (coefficient of variation typically ≈3 %) are produced, i.e. variations of the volume and any related errors due to polydispersity can be neglected. Initially, DEs with a diameter of 25 μm and a volume of ≈8.2 pL were tested. After optimization of the chip design, the final diameter and volume were reduced to 15 μm and ≈1.8 pL respectively. This droplet volume is ideal as it allows for quick accumulation of the product and fits perfectly in the FACS workflow. Moreover, since only one *E. coli* is encapsulated per droplet, the smaller volume of the droplet leads to a higher concentration of the ArM. This feature is reflected in an increased conversion and thus a more reliable signal to noise ratio. To produce DEs containing the reaction mixture, the following protocol was applied: a solution of *E. coli* previously incubated with cofactor **1** in phosphate buffered saline (PBS) was introduced into the chip via a first inlet. Since the biotinylated cofactor consists of a CpRu‐precursor and a biotinylated ligand (Figure 1), both stock solutions were prepared in dimethylformamide (DMF) to ensure their dissolution and pre‐incubated with *E. coli* (expressing Sav in the periplasm) in PBS (+0.5 % DMF) (for details, see Supporting Information). The high affinity of biotin for Sav (*K*_d_≈10^−14^ M) ensures that the biotinylated hydrophobic cofactor remains compartmentalized in the periplasm of the *E. coli* after binding to Sav and does not diffuse out of the droplet. The solution of the substrate **2** in PBS was added via a second inlet. The use of two inner aqueous (IA) phase inlets shields the *E. coli* and ADAse from the substrate **2** until on‐chip encapsulation. The PDMS chip, the encapsulation conditions and the dilution of the cell suspension were optimized to afford ≈77 % empty droplets, ≈20 % droplets containing one cell, and the remaining 2.6 % containing two or more cells per droplet. Although there are reported methods to improve this ratio further,^[41]^ the throughput of both droplet production and FACS allowed us to conveniently work with this ratio.

*E. coli* can survive in DEs for at least 10 hours, as previously shown by growth experiments and fluorogenic assays.^[35]^ However, the overexpression of Sav leads to premature cell‐death, as it depletes the cell from biotin.^[42]^ However, as the phenotype‐genotype linkage is maintained within each droplet, cell survival is not required to enable deconvolution of the best Sav mutants by NGS.

After collection and incubation of the DEs off‐chip, the fluorescence in the inner aqueous phase was quantified by FACS. The DEs were first gated based on forward‐ and side‐scatter profiles to separate them from oil droplets and from non‐homogeneous DEs, e.g., those containing two inner aqueous phase droplets (Figure 3b). Empty DEs could be further sorted based on the absence of a fluorescent signal from mNectarine. To quantify the coumarin **3** FI and stability, different DE populations were prepared with substrate **2** (500 μM) and **3** in three different concentrations (5, 50 and 500 μM). Flow cytometry analysis of these populations one and 24 h after encapsulation confirmed the stability of both the substrate and product, with minimal leakage in the course of 24 h (Figure 3c). The presence of a sulfonate group on both substrate **2** and product **3** minimizes their diffusion into the hydrophobic oil‐phase. Furthermore, these results demonstrate that the three product concentrations can be clearly distinguished from the background fluorescence of substrate **2**. We also investigated whether the presence of *E. coli* cells affects the stability of substrate **2** or the catalytic activity of the ADAse. In particular, we co‐encapsulated (i) *E. coli* cells harboring wildtype streptavidin (wt‐Sav) in their periplasm with substrate **2** (500 μM) and (ii) *E. coli* cells lacking the plasmid for Sav expression (previously incubated with 5 μM of **1**) together with the substrate **2** (500 μM). In both cases, DEs containing cells were compared with DEs without cells after 0 h and 10 h of incubation. In the case of (i), the fluorescence determined for DEs with and without cells was within the same range and remained constant over time (Figure 3d). For scenario (ii), both DE populations again displayed very similar fluorescence intensity initially and similar increases in fluorescence over time as more substrate **2** was converted into product **3** (Figure 3e).

**Figure 4:**
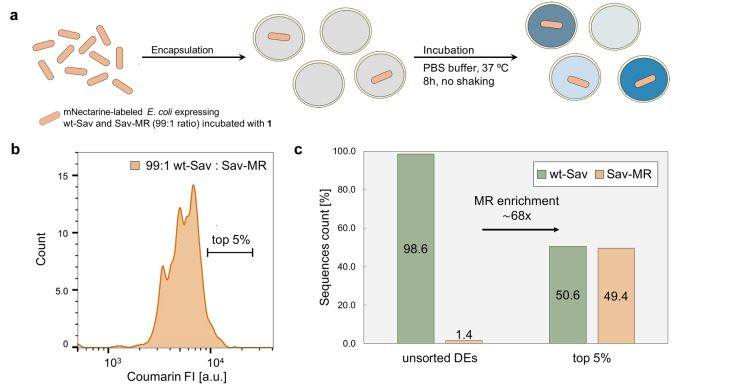
Control experiments with wt‐Sav and Sav‐MR. a) mNectarine‐labeled *E. coli* expressing wt‐Sav and Sav‐MR (99 : 1 ratio) were pre‐incubated with cofactor **1** (5 μM) in PBS buffer, encapsulated in DEs with substrate 2 (500 μM), and incubated at 37 °C for 8 h with no shaking. b) The coumarin FI distribution of the resulting DEs and the top 5 % gate selected for analysis by FACS. c) Enrichment of Sav‐MR with respect to wt‐Sav in the top 5 % of the population determined by NGS.

Next, the screening method was validated by conducting an enrichment experiment wherein mNectarine‐labeled *E. coli* expressing wt‐Sav or a known variant Sav‐MR were combined in a 99 : 1 ratio, incubated with cofactor **1**, and then encapsulated in DEs along with the substrate **2** as described earlier (Figure 4a). The variant Sav‐MR bears two mutations at S112M and K121R, which leads to improved ADAse activity.^[23]^ After incubation, the DE sample was sorted by FACS and the population of droplets with highest coumarin FI was collected (Figure 4b). After plasmid extraction, NGS was carried out to determine the enrichment of Sav‐MR in the top 5 % gate. The enrichment factor was determined by applying the following formula: (Sav‐MR_top5 %_/wt‐Sav_top5 %_)/(Sav‐MR_unsorted_)/wt‐Sav_unsorted_). Sequence analysis revealed a 68‐fold enrichment of Sav‐MR relative to the unsorted DEs sample, thereby validating the reliability of the assay (Figure 4c).

### Validation of the Assay by Screening a 400‐Variant Library

**Figure 5:**
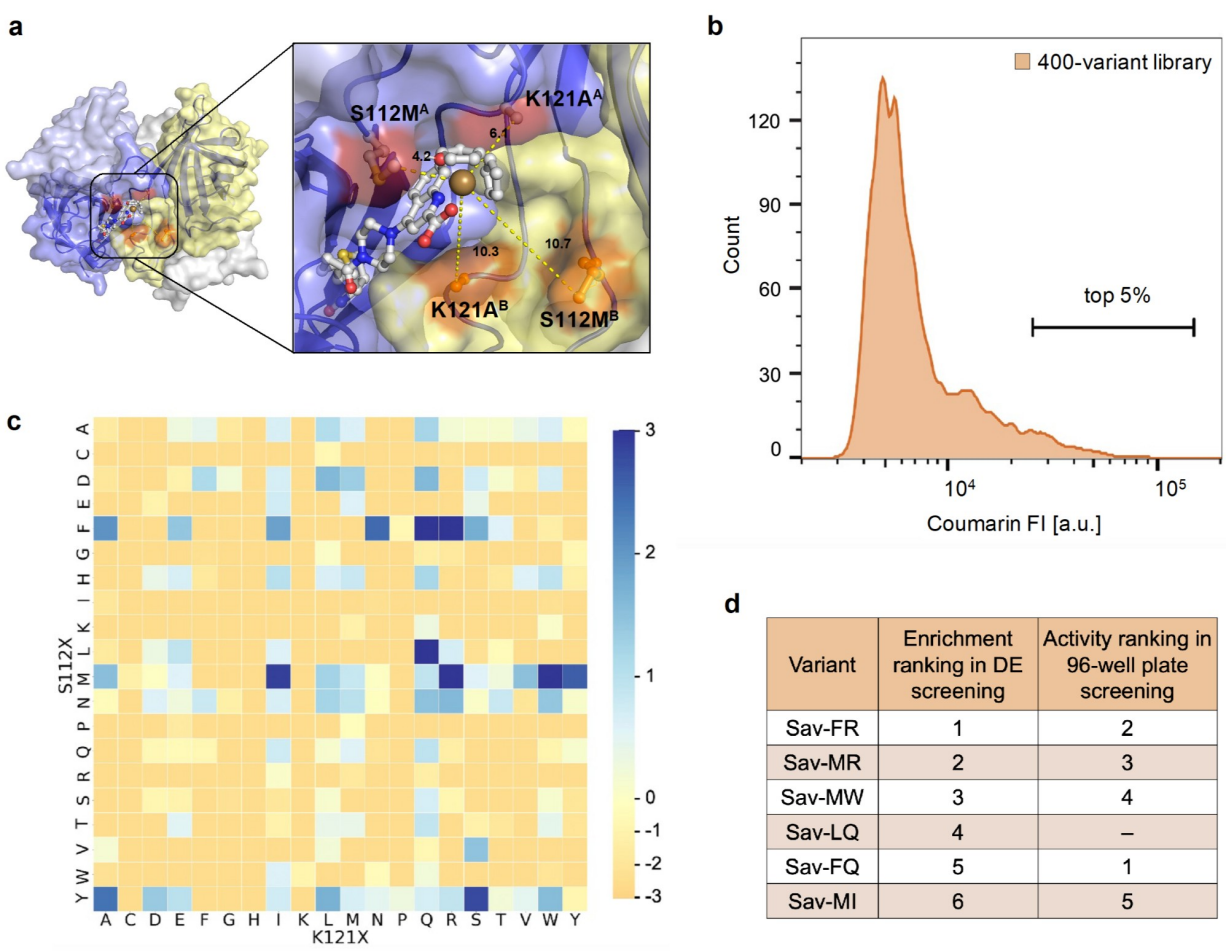
Screening of a 400‐variant library in DEs and comparison with a 96‐well plate assay. a) Crystal structure of the ADAse **1**⋅Sav S112M‐K121A (PDB‐ID: 6FH8).^[22]^ The four Sav monomers are displayed as transparent surfaces in blue, pale blue, yellow and pale yellow, respectively. The mutated positions in Sav^A^ (S112M^A^ and K121A^A^, red) and the positions facing the active site in Sav^B^ (S112M^B^ and K121A^B^, orange) are highlighted and labelled. The biotinylated ruthenium complex **1** is shown using a ball and stick representation and is anchored in Sav^A^ (blue). Dark yellow, red, blue, grey, yellow, and white spheres represent Ru, O, N, C, S, and H atoms, respectively. b) Coumarin FI distribution of DEs containing the library. The top 5 % gate chosen for sorting is highlighted. c) Visual representation of the enrichment of the 400 mutants over the unsorted sample in the top 5 % of the DE population. Amino acids at position K121 are represented on the x‐axis, and amino acids at position S112 are represented on the y‐axis. The normalized enrichment values of the respective mutants over their occurrence in the unsorted sample are displayed on a logarithmic scale. Blue: positive enrichment (values>0), orange: negative enrichment (values<0). d) Comparison of the top six mutants identified from DE screening and 96‐well plate screening.

The method was next applied to the screening of a 400‐variant library of Sav double mutants bearing mutations at positions S112 and K121. This library was previously screened using an automated 96‐well plate assay, which revealed six variants with activities ≥12‐fold higher than wt‐Sav for the deallylation of **2**, namely Sav‐FQ, ‐FR, ‐MR, ‐MW, ‐MI and ‐AW (Figure 5b, Supporting Information Tables 1 and 2).^[23]^ After preparation of the DE sample and incubation, the top 5 % of the DEs with highest coumarin FI were sorted by FACS (Figure 5b). Plasmids were extracted from the sorted and unsorted populations and analyzed by NGS to determine the enrichment factor of the 5 % gate. Notably, of the six variants previously identified using the automated 96‐well plate assay, five (excluding Sav‐AW) were identified by NGS as having the highest enrichment in the top 5 % gate (Figure 5c and Supporting Information Figure 2). Additional variants including Sav‐LQ and Sav‐MY were also enriched in the top 5 % gate relative to the unsorted sample. We confirmed the reproducibility of these results by repeating the screening using a biological replicate. A comparison of the 20 most enriched variants determined from both data sets revealed an overlap of 75 % and a good correlation for the enrichment of all mutants (Supporting Information Figure 3, R^2^=0.88). These results highlight the versatility of the DE screening approach and its potential for the time‐efficient screening of libraries with minimal reagent consumption. Indeed, the whole screening process—from the transformation of the 400‐variant library into *E. coli* cells to the NGS data analysis—was achieved within one week, and required only 12.5 pmol of cofactor/variant analyzed. In contrast, traditional 96‐well plate screening of a library of this size requires 2 nmol of cofactor/variant, which is more than a 100‐fold increase in reagent consumption.

## Conclusion

Using droplet microfluidics for the high‐throughput encapsulation of live *E. coli* and readily available FACS instrumentation, we have developed a straightforward method for the rapid screening of ArMs, using a deallylase based on the biotin‐streptavidin technology as a model system. We demonstrated the potential of this approach by performing an enrichment experiment with a model library of variants, housed in the periplasm of *E. coli*, and achieving more than 68‐fold enrichment of the most active variant. We further screened a library of 400 variants using the same periplasmic approach and identified five out of the six hits previously identified using a MTP screening protocol.^[23]^ Significantly, this method requires far less time and fewer reagents than standard methods, and the screening of much larger libraries (>500 000 variants) would not require considerably more time for the encapsulation, incubation and sorting (steps 2–4 in Figure 1b).

In summary, we have demonstrated that a single round of screening enables us to enrich moderately improved variants (i.e., ≈15‐fold improved activity vs WT ADAse^[23]^) from a medium‐sized library of ArMs, compartmentalized in the periplasm of *E. coli*. This proof‐of‐concept lays the groundwork for future microfluidics‐based directed evolution campaigns of (artificial) enzymes that afford a fluorescent (by‐)product. We anticipate that the microfluidic platform presented herein will render ultra‐high throughput screening of enzymatic activity accessible to virtually any research laboratory with a minimal capital investment (<30 k$) and limited microfluidic expertise.

## Conflict of interest

The authors declare no conflict of interest.

1

## Supporting information

As a service to our authors and readers, this journal provides supporting information supplied by the authors. Such materials are peer reviewed and may be re‐organized for online delivery, but are not copy‐edited or typeset. Technical support issues arising from supporting information (other than missing files) should be addressed to the authors.

Supporting Information

## Data Availability

The data that support the findings of this study are available in the Supporting Information of this article.

## References

[1] K. Chen , F. H. Arnold , Proc. Natl. Acad. Sci. USA 1993, 90, 5618–5622. 8516309 10.1073/pnas.90.12.5618 PMC46772

[2] L. Giger , S. Caner , R. Obexer , P. Kast , D. Baker , N. Ban , D. Hilvert , Nat. Chem. Biol. 2013, 9, 494–498. 23748672 10.1038/nchembio.1276 PMC3720730

[3] M. T. Reetz , A. Zonta , K. Schimossek , K. Liebeton , K. E. Jaeger , Angew. Chem. Int. Ed. Engl. 1997, 36, 2830–2832;

[4] I. V. Pavlidis , M. S. Weiß , M. Genz , P. Spurr , S. P. Hanlon , H. Iding , U. T. Bornscheuer , Nat. Chem. 2016, 8, 1076–1082. 27768108 10.1038/nchem.2578

[5] D. S. Macdonald , X. Garrabou , C. Klaus , R. Verez , T. Mori , D. Hilvert , J. Am. Chem. Soc. 2020, 142, 10250–10254. 32427470 10.1021/jacs.0c02351

[6] S. Kille , C. G. Acevedo-Rocha , L. P. Parra , Z. G. Zhang , D. J. Opperman , M. T. Reetz , J. P. Acevedo , ACS Synth. Biol. 2013, 2, 83–92. 23656371 10.1021/sb300037w

[7] A. Li , G. Qu , Z. Sun , M. T. Reetz , ACS Catal. 2019, 9, 7769–7778.

[8] F. Schwizer , Y. Okamoto , T. Heinisch , Y. Gu , M. M. Pellizzoni , V. Lebrun , R. Reuter , V. Köhler , J. C. Lewis , T. R. Ward , Chem. Rev. 2018, 118, 142–231. 28714313 10.1021/acs.chemrev.7b00014

[9] M. Jeschek , R. Reuter , T. Heinisch , C. Trindler , J. Klehr , S. Panke , T. R. Ward , Nature 2016, 537, 661–665. 27571282 10.1038/nature19114

[10] A. D. Liang , J. Serrano-Plana , R. L. Peterson , T. R. Ward , Acc. Chem. Res. 2019, 52, 585–595. 30735358 10.1021/acs.accounts.8b00618 PMC6427477

[11] J. G. Rebelein , Y. Cotelle , B. Garabedian , T. R. Ward , ACS Catal. 2019, 9, 4173–4178. 31080690 10.1021/acscatal.9b01006 PMC6503580

[12] Z. Liu , F. H. Arnold , Curr. Opin. Biotechnol. 2021, 69, 43–51. 33370622 10.1016/j.copbio.2020.12.005 PMC8225731

[13] K. Oohora , A. Onoda , T. Hayashi , Acc. Chem. Res. 2019, 52, 945–954. 30933477 10.1021/acs.accounts.8b00676

[14] S. N. Natoli , J. F. Hartwig , Acc. Chem. Res. 2019, 52, 326–335. 30693758 10.1021/acs.accounts.8b00586 PMC11620731

[15] J. C. Lewis , Acc. Chem. Res. 2019, 52, 576–584. 30830755 10.1021/acs.accounts.8b00625

[16] A. Lombardi , F. Pirro , O. Maglio , M. Chino , W. F. DeGrado , Acc. Chem. Res. 2019, 52, 1148–1159. 30973707 10.1021/acs.accounts.8b00674 PMC7362765

[17] G. Roelfes , Acc. Chem. Res. 2019, 52, 545–556. 30794372 10.1021/acs.accounts.9b00004 PMC6427492

[18] C. S. Mocny , V. L. Pecoraro , Acc. Chem. Res. 2015, 48, 2388–2396. 26237119 10.1021/acs.accounts.5b00175 PMC5257248

[19] C. Streu , E. Meggers , Angew. Chem. Int. Ed. 2006, 45, 5645–5648; 10.1002/anie.200601752 16856188

[20] T. Völker , F. Dempwolff , P. L. Graumann , E. Meggers , Angew. Chem. Int. Ed. 2014, 53, 10536–10540; 10.1002/anie.201404547 25138780

[21] T. Völker , E. Meggers , ChemBioChem 2017, 18, 1083–1086. 28425643 10.1002/cbic.201700168

[22] T. Heinisch , F. Schwizer , B. Garabedian , E. Csibra , M. Jeschek , J. Vallapurackal , V. B. Pinheiro , P. Marlière , S. Panke , T. R. Ward , Chem. Sci. 2018, 9, 5383–5388. 30079176 10.1039/c8sc00484f PMC6048633

[23] T. Vornholt , F. Christoffel , M. M. Pellizzoni , S. Panke , T. R. Ward , M. Jeschek , Sci. Adv. 2021, 7, 4208–4230. 10.1126/sciadv.abe4208 PMC10964965 33523952

[24] U. Markel , K. D. Essani , V. Besirlioglu , J. Schiffels , W. R. Streit , U. Schwaneberg , Chem. Soc. Rev. 2020, 49, 233–262. 31815263 10.1039/c8cs00981c

[25] A. Stucki , J. Vallapurackal , T. R. Ward , P. S. Dittrich , Angew. Chem. Int. Ed. 2021, 60, 24368–24387; 10.1002/anie.202016154 PMC8596820 33539653

[26] J. J. Agresti , E. Antipov , A. R. Abate , K. Ahn , A. C. Rowat , J. C. Baret , M. Marquez , A. M. Klibanov , A. D. Griffiths , D. A. Weitz , Proc. Natl. Acad. Sci. USA 2010, 107, 4004–4009. 20142500 10.1073/pnas.0910781107 PMC2840095

[27] B. Kintses , C. Hein , M. F. Mohamed , M. Fischlechner , F. Courtois , C. Lainé , F. Hollfelder , Chem. Biol. 2012, 19, 1001–1009. 22921067 10.1016/j.chembiol.2012.06.009

[28] R. Obexer , M. Pott , C. Zeymer , A. D. Griffiths , D. Hilvert , Protein Eng. Des. Sel. 2016, 29, 355–366. 27542390 10.1093/protein/gzw032

[29] J. M. Holstein , C. Gylstorff , F. Hollfelder , ACS Synth. Biol. 2021, 10, 252–257. 33502841 10.1021/acssynbio.0c00538 PMC7901014

[30] R. Obexer , A. Godina , X. Garrabou , P. R. E. Mittl , D. Baker , A. D. Griffiths , D. Hilvert , Nat. Chem. 2017, 9, 50–56. 27995916 10.1038/nchem.2596

[31] D. Vallejo , A. Nikoomanzar , B. M. Paegel , J. C. Chaput , ACS Synth. Biol. 2019, 8, 1430–1440. 31120731 10.1021/acssynbio.9b00103 PMC6942126

[32] M. Fischlechner , Y. Schaerli , M. F. Mohamed , S. Patil , C. Abell , F. Hollfelder , Nat. Chem. 2014, 6, 791–796. 25143214 10.1038/nchem.1996

[33] K. K. Brower , C. Carswell-Crumpton , S. Klemm , B. Cruz , G. Kim , S. G. K. Calhoun , P. M. Fordyce , Lab Chip 2020, 20, 2062–2074. 32417874 10.1039/d0lc00261e PMC7670282

[34] K. K. Brower , M. Khariton , P. H. Suzuki , C. Still , G. Kim , S. G. K. Calhoun , L. S. Qi , B. Wang , P. M. Fordyce , Anal. Chem. 2020, 92, 13262–13270. 32900183 10.1021/acs.analchem.0c02499 PMC7670281

[35] A. Stucki , P. Jusková , N. Nuti , S. Schmitt , P. S. Dittrich , Small Methods 2021, 5, 2100331. 10.1002/smtd.202100331 34927870

[36] K. Bernath , M. Hai , E. Mastrobattista , A. D. Griffiths , S. Magdassi , D. S. Tawfik , Anal. Biochem. 2004, 325, 151–157. 14715296 10.1016/j.ab.2003.10.005

[37] E. Mastrobattista , V. Taly , E. Chanudet , P. Treacy , B. T. Kelly , A. D. Griffiths , Chem. Biol. 2005, 12, 1291–1300. 16356846 10.1016/j.chembiol.2005.09.016

[38] S. S. Terekhov , I. V. Smirnov , A. V. Stepanova , T. V. Bobik , Y. A. Mokrushina , N. A. Ponomarenko , A. A. Belogurov , M. P. Rubtsova , O. V. Kartseva , M. O. Gomzikova , A. A. Moskovtsev , A. S. Bukatin , M. V. Dubina , E. S. Kostryukova , V. V. Babenko , M. T. Vakhitova , A. I. Manolov , M. V. Malakhova , M. A. Kornienko , A. V. Tyakht , A. A. Vanyushkina , E. N. Ilina , P. Masson , A. G. Gabibov , S. Altman , Proc. Natl. Acad. Sci. USA 2017, 114, 2550–2555. 28202731 10.1073/pnas.1621226114 PMC5347554

[39] A. Zinchenko , S. R. A. Devenish , B. Kintses , P. Y. Colin , M. Fischlechner , F. Hollfelder , Anal. Chem. 2014, 86, 2526–2533. 24517505 10.1021/ac403585p PMC3952496

[40] A. C. Larsen , M. R. Dunn , A. Hatch , S. P. Sau , C. Youngbull , J. C. Chaput , Nat. Commun. 2016, 7, 11235. 27044725 10.1038/ncomms11235 PMC4822039

[41] J. F. Edd , D. di Carlo , K. J. Humphry , S. Köster , D. Irimia , D. A. Weitz , M. Toner , Lab Chip 2008, 8, 1262–1264. 18651066 10.1039/b805456h PMC2570196

[42] M. Jeschek , M. O. Bahls , V. Schneider , P. Marlière , T. R. Ward , S. Panke , Metab. Eng. 2017, 40, 33–40. 28062280 10.1016/j.ymben.2016.12.013

