# Supplementary material for: Ultrahigh‐Throughput Screening of an Artificial Metalloenzyme using Double Emulsions
Source: Angew Chem Int Ed Engl. 2022 Oct 27;61(48):e202207328. doi: 10.1002/anie.202207328 (PMC9828110; doi:10.1002/anie.202207328)
Supplement: Supplementary file 1 — Supporting Information [file ANIE-61-0-s001.pdf]

Angewandte Chemie
Eine Zeitschrift der Gesellschaft Deutscher Chemiker

Supporting Information

Ultrahigh-Throughput Screening of an Artificial Metalloenzyme using Double Emulsions

J. Vallapurackal, A. Stucki, A. D. Liang, J. Klehr, P. S. Dittrich, T. R. Ward**

Experimental Section

Materials and Methods

Materials. Unless otherwise stated, PBS was purchased from Bioconcept. 1*H*,1*H*,2*H*,2*H*-perfluorooctanol was bought from Fluorochem. Tris-(acetonitrile)-cyclopentadienylruthenium(II)-hexafluorophosphate was purchased from Sigma Aldrich. Water was purified with a Milli-Q-system (Millipore). Antibiotics were purchased from Applichem GmbH. All enzymes, the Monarch plasmid extraction kit, the Monarch PCR and DNA clean-up kit, and the Gibson assembly master mix, were purchased from New England BioLabs. Magnetic beads used for DNA purification were AMPure XP purchased from Beckman-Coulter Life Sciences. Sodium dodecyl sulfate (SDS) was bought from abcr. Poly(dimethylsiloxane) (PDMS, Sylgard 184) was purchased from Dow Corning. Hydrofluoroether (HFE 7500) with 5% 008-FluoroSurfactant was purchased from RAN Biotechnologies, Inc. All supplies for Nanopore sequencing were purchased from Oxford Nanopore Technologies.

The bacterial strain Top10 DE3 was designed and generously provided by the Panke laboratory[1]. Chemically competent cells (prepared according to the RbCl-method following the Hanahan protocol) and electrocompetent cells bearing the mNectarine plasmid were prepared in the laboratory[2].

The pET30b vector (Supplementary Fig. 4) was used for the generation of the mutant library. The pUA66 vector (Supplementary Fig. 5) was used for mNectarine[3]. Primers for NGS were designed individually and were synthesized at IDT technologies or Microsynth AG (Supplementary Table 5).

Methods. For all biological experiments, the equipment was sterilised (121 °C, 20 min). PCR reactions were performed with an Eppendorf Mastercycler Gradient following a general protocol (Supplementary Tables 3 and 4). Agarose and SDS gel electrophoresis chambers and the MicroPulsor Electroporator were purchased from Bio-Rad Laboratories Inc. The gels were visualized with the software Quantity One. DNA concentrations were measured with NanoDrop1000 or the Advanced Analytical 12-capillary Fragment Analyzer from Agilent. Fluorescence scans of individual substances were conducted using a Tecan Infinite M1000 Pro. Large scale protein purification was done using ÄKTA prime Fast Liquid Protein Chromatography System (GE Healthcare). DE droplets were analyzed on a BD LSR Fortessa SORP (Special Order Research Product) and sorted on a BD FACSAria™ II cell sorter. NGS was carried out on an Illumina NextSeq 500 platform.

For the microfluidics assay, neMESYS syringe pumps (Cetoni) and 1 mL syringes (VWR, BD Plastipak Luer-lock) were used to introduce the solutions into the microfluidic chip. Polytetrafluoroethylen (PTFE) tubing (ID = 0.56 mm, Adtech Polymer Engineering™), precision dispenser needles (23 gauge, Metcal) and metal pins (New England Small Tube, NE-1310-03, 0.025″ OD × .013″ ID x 1.00″ length) to connect the syringes to the chip. To control the chip coating with polyvinyl alcohol (PVA), we used a MFCS-8C pressure control unit (Fluigent).

Library Preparation.

400-variants library: pET30b_Sav_library. A glycerol stock containing *E. coli* cells with the 400-mutant library at positions S112 and K121 was grown overnight and the plasmid extracted. The purified plasmid (~100 ng) was transformed into electrocompetent Top10(DE3) cells (50 µL), containing the plasmid pSC101 with mNectarine encoded. Electroshock was applied using the MicroPulser electroporator by Bio-Rad Laboratories, Inc. Immediately after the electroshock, SOC-medium (450 µL) was added, the reaction transferred to a sterile Eppendorf tube and incubated at 37 °C for 40-60 min. The transformation was split into four equal parts and plated on 12 cm × 12 cm LB-agar plates supplemented with kanamycin and chloramphenicol and incubated overnight at 37 °C. All plates were scraped by the addition of LB-medium (2 mL per plate) and the combined cell suspension was aliquoted. 100 µL cell suspension was mixed with 100 µL of a glycerol stock solution (50%) to obtain 20 aliquots with a ~25 % final glycerol concentration. The glycerol stocks were immediately frozen in liquid N_2 and finally stored at −80 °C. One whole such aliquot was used for the inoculation of cultures for the screening assay.

mNectarine plasmid. The plasmid for the constitutive expression of mNectarine is based on pUA66[3]. The kanamycin resistance cassette was exchanged for a chloramphenicol resistance by amplifying insert and backbone with compatible overhangs and assembling them by Gibson Assembly. Likewise, GFP was exchanged for mNectarine with promoter BBa_J23111 and ribosome binding site BBa_B0030. This plasmid was generously provided by Tobias Vornholt and Markus Jeschek (ETHZ, D-BSSE).

Substrate and product preparation. Triethylammonium (7-(((allyloxy)carbonyl)amino)-2-oxo-2H-chromen-4-yl)methanesulfonate **2** and triethylammonium (7-amino-2-oxo-2H-chromen-4-yl)methanesulfonate **3** were synthesized as previously reported[4,5]. Stock solutions of substrate **2** (10 mM) and product **3** (10 mM) were prepared in freshly filtered PBS (0.2 µm filter), aliquoted in 60 µL samples to avoid multiple cycles of freezing and thawing and stored at −20 °C for further use.

Cofactor preparation. The cofactor was prepared *in situ* by mixing a solution of $[CpRu(MeCN)_3]PF_6$ (2 mM in degassed *N*,*N*-dimethylformamide) and a solution of the biotinylated ligand (2 mM in degassed *N*,*N*-dimethylformamide) in a 1:1 ratio inside a N_2 glovebox. The biotinylated ligand was synthesized using a previously reported procedure[6]. A stock solution of cofactor **1** (1 mM in *N*,*N*-dimethylformamide) was used outside the glovebox after incubation for 5-10 min at room temperature.

General protocol for Sav expression for the screening. A preculture of LB medium (5 mL) supplemented with chloramphenicol (32 mg/mL) and kanamycin (50 mg/mL) was inoculated with the previously prepared glycerol stock of the library of interest and incubated for 8 h at 37 °C and 300 rpm. A culture of LB medium (25 mL) supplemented with chloramphenicol (32 mg/mL) and kanamycin (50

mg/mL) in a shaking flask (250 mL) was inoculated with the preculture to a starting OD_{600} = 0.05 and incubated for ~1-2 h at 37 °C and 300 rpm (until an OD_{600} = 0.5-0.8 was reached). Sav expression was induced by the addition of IPTG (50 µM final concentration) and expression was performed overnight at 25 °C and 300 rpm. One sample (1 mL) of cell culture with an OD_{600} = 0.20 was prepared and centrifuged (5 min; 17000 g). The supernatant was discarded and the pellet was resuspended in PBS (990 µL, pH 7.4). To this cell suspension, the cofactor **1** stock solution (10 µL, 1 mM, 10 µM final concentration) was added to afford the cell-cofactor mixture for the droplet production.

Microfluidic set-up.

Microfluidic platform fabrication. The devices were produced according to a protocol described previously[7]. Briefly, the microfluidic chips were produced using a SU-8 master mold on which a mixture of PDMS and curing agent (ratio 10:1) was poured. The wafer was cured at 80 °C for 3 h. After punching inlets and outlets with a biopsy puncher (diameter 0.5 mm), the chips were plasma bonded to PDMS-coated glass slides. The device has four inlets for the outer aqueous phase (OA), the oil phase (OP) and the inner aqueous phases (IA1 and IA2), and one outlet. To allow for DE formation, a 2.5 % poly(vinyl alcohol) (PVA) solution was used to coat the OA and outlet channels with a hydrophilic layer, according to a protocol previously described by Deshpande *et al*[8].

Microfluidic assay. DE formation was achieved using flow rates between 0.5 and 5 µL/min for all solutions (IA1, IA2, OA, OP) and was monitored on an inverted microscope (IX71, Olympus) with a high-speed camera (Phantom VEO, Vision Research). In a typical experiment, the solutions contained the following components. OA: PBS with 5 g L^{-1} SDS, OP: HFE 7500 with 2% 008-FluoroSurfactant, IA1: PBS with bacteria (OD_{600} = 0.2) and cofactor (10 µM) and IA2: PBS with substrate (1000 µM). The DEs were collected in a 1.5 mL Eppendorf tube *via* a short piece of PTFE tubing connected to the outlet.

DE incubation and FACS. The DEs were incubated statically in collection tubes in an oven at 37 ºC for at least 3 h. For measurements on the flow cytometer, 0.5 µL of DE solution were added to 300 µL of OA (PBS with 5 g/L SDS), agitated manually and loaded on a BD LSR Fortessa SORP (Special Order Research Product). The DEs were gated by size based on forward and side scatter profiles. The DEs were then gated for singlets using bivariate plots of DEs side scatter height versus area, followed by gating for mNectarine fluorescence (mNectarine: λ_{ex} = 558 nm, λ_{em} = 578 nm; 561 nm laser configuration, bandpass filter 610/20) to determine the presence/absence of cells. Finally, the coumarin fluorescence was determined (coumarin: λ_{ex} = 395 nm, λ_{em} = 460 nm; 405 nm laser configuration, bandpass filter 450/50). For sorting experiments, 10 µL of DE solution were added to 600 µL of OA (PBS with 5 g L^{-1} SDS), agitated manually and loaded on a BD FACS Aria™ II cell sorter. This operation was repeated every time the sample tube was empty. During the FACS measurements, the samples were regularly agitated by the operator to prevent the DEs from settling. The DEs were gated as described above and the top 5% of the coumarin **3** peak was sorted into a collection.

DNA sequencing

Plasmid recovery. The sorted samples were spun down, and the supernatant (PBS + 0.5 % SDS) was removed. The droplets were then resuspended in the resuspension buffer (B1, 50 µL) of the Monarch plasmid extraction kit. Additionally, perfluorooctanol (12 µL) was added and the suspension was incubated at 50 °C for 20-40 min and 300 rpm. Next, lysis buffer (B2, 50 µL) was added and incubated at room temperature for 1 min. Finally, the neutralization buffer (B3, 100 µL) was added and incubated at room temperature for 2 min. The subsequent steps were carried out following the protocol from the manufacturer (Monarch PCR clean-up kit by NEB), and elution was performed twice with 6 µL of mQ H_2O (heated to 70 °C).

NGS measurements. Plasmid DNA (extracted from the five sorted populations and the parent) was amplified using the NGS primers containing the barcodes, adapters and indices for sequencing (Supplementary Table 5). The PCR products were purified by agarose gel electrophoresis and the concentration and purity were determined by capillary electrophoresis. The different samples were pooled according to the measured concentrations to have an equimolar input for the NGS run. The sequencing was carried out with a NextSeq Mid Output v2 kit (300 cycles, PE 2× 150) spiked with additional 20% PhiX. Primary data analyses were performed with Illumina RTA version 2.4.11 and bcl2fastq v2.20.0.422.

Data evaluation.

Processing of NGS data: We used in-house bash and R scripts to analyze the NGS data. Fastq files containing the forward and reverse reads were obtained following NGS. The reads were extracted and paired. The reads were filtered using the 24-bp fixed region located between position 112 and position 121 (Supplementary Fig. 4), allowing for a maximum of three mismatches. The target fragments were attributed to each sample using their unique barcode (Supplementary Table 5). The mutations at positions 112 and 121 were identified by retrieving the 3 nucleotides before and after the fixed sequence.

Supplementary Figures

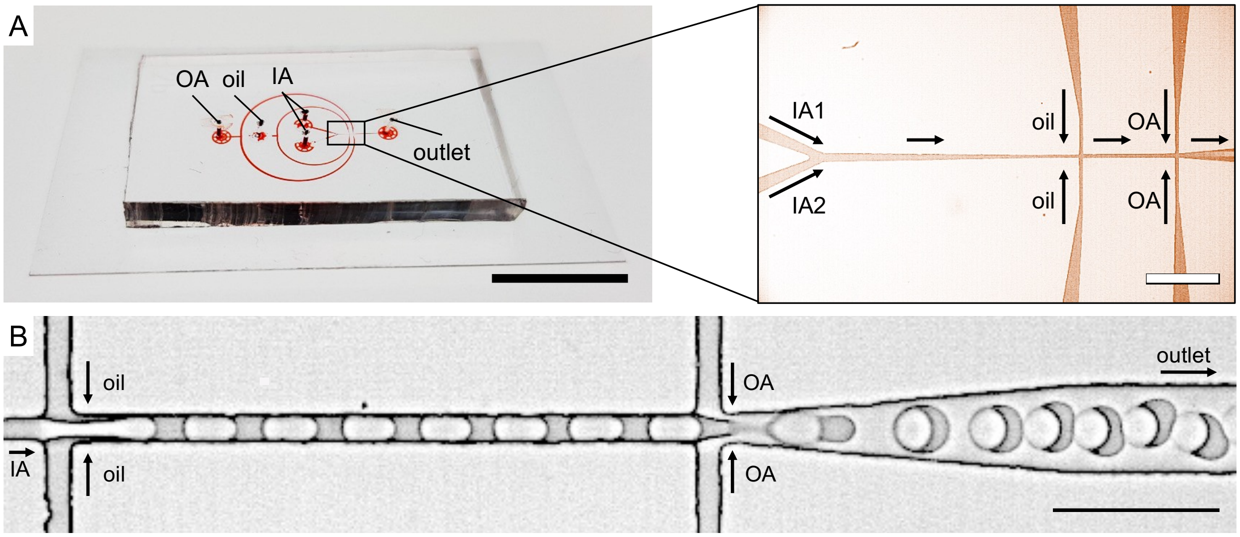

Supplementary Figure 1. Microfluidic chip for the double emulsion production. A**,** Photograph and micrograph of the microfluidic device designed for DE formation. The device has channels with a height of 11 µm and contains 4 inlets: one inlet for the outer aqueous phase (OA), one inlet for the oil (oil) and two inlets for the inner aqueous phases (IA). Black scale bar: 1 cm. White scale bar: 200 µm. B**,** Micrograph of DE formation. Monodisperse DEs co-encapsulating *E. coli* cells, cofactor **1** and substrate **2** are produced using a PDMS-based microfluidic device. Scale bar: 50 µm.

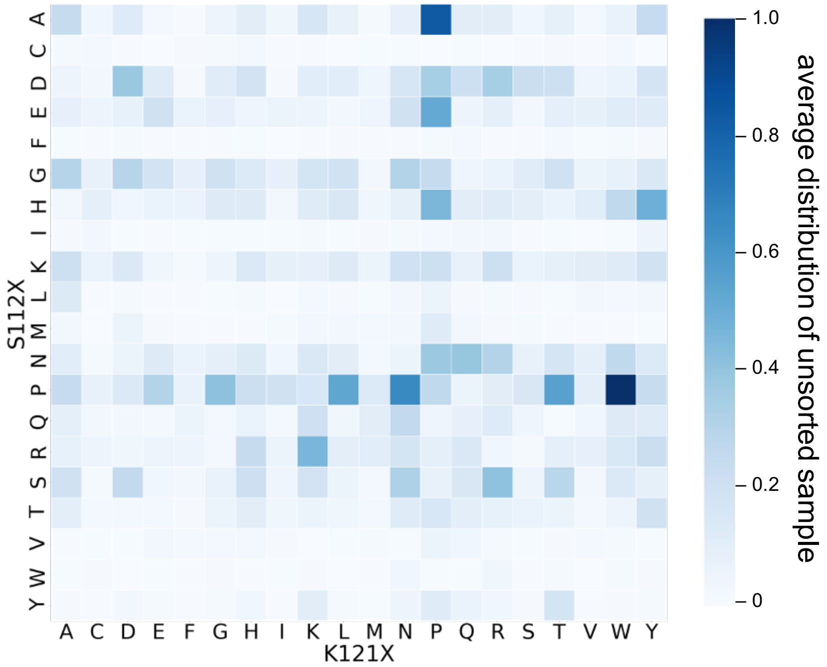

Supplementary Figure 2. Screening of a 400-variant library in DEs. Relative distribution of the 400 mutants in the unsorted sample determined by NGS. *x* axis: amino acids at position K121, *y* axis: amino acids at position S112. Displayed are relative occurrences of the respective mutants in relation to the overall reads in the whole sample determined by NGS.

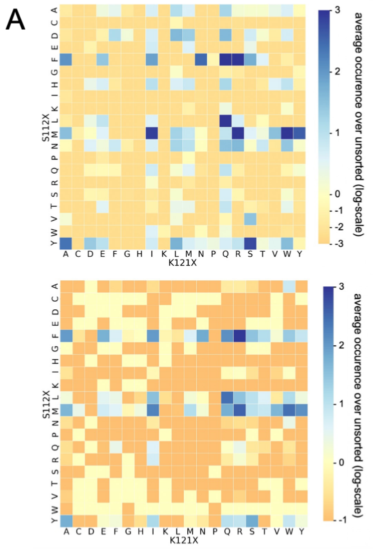

B

Rank	Data Set 1 Mutants	Data Set 1 Enrichment	Biological Replicate Mutants	Biological Replicate Enrichment
1	FR	749.6	FR	917.2
2	MR	358.7	MR	360.2
3	MW	123.5	LQ	263.1
4	LQ	42.5	MW	259.1
5	FQ	17.3	MI	174.5
6	MI	14.4	MY	125.2
7	YS	13.2	FQ	121.8
8	MY	11.0	FI	94.8
9	FN	10.1	FA	93.2
10	YA	9.2	FN	67.2
11	FA	6.9	YS	64.0
12	FI	5.9	MQ	59.7
13	FS	5.2	YA	57.0
14	YL	4.9	FE	53.1
15	DL	4.6	MV	47.3
16	DQ	4.5	MA	45.9
17	NR	4.5	LI	34.6
18	YW	4.4	LR	31.6
19	MA	4.2	FS	30.9
20	MV	4.2	FW	23.7

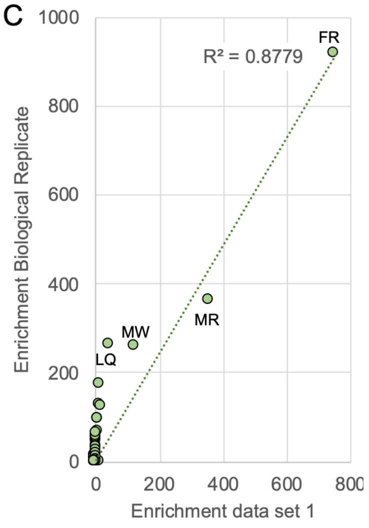

Supplementary Figure 3. Screening validation and data reproducibility for the 400-variant library. **A** Heat map comparison between enrichments found for data set 1 (top) and biological replicate (bottom). **B** Enrichment values for the top 20 mutants in data set 1 and its biological replicate. Green: mutants found in the top 20 of both screenings at the same rank. Blue: mutants found in the top 20 of both screenings. **C** Correlation curve between the enrichment values found for data set 1 and its biological replicate.

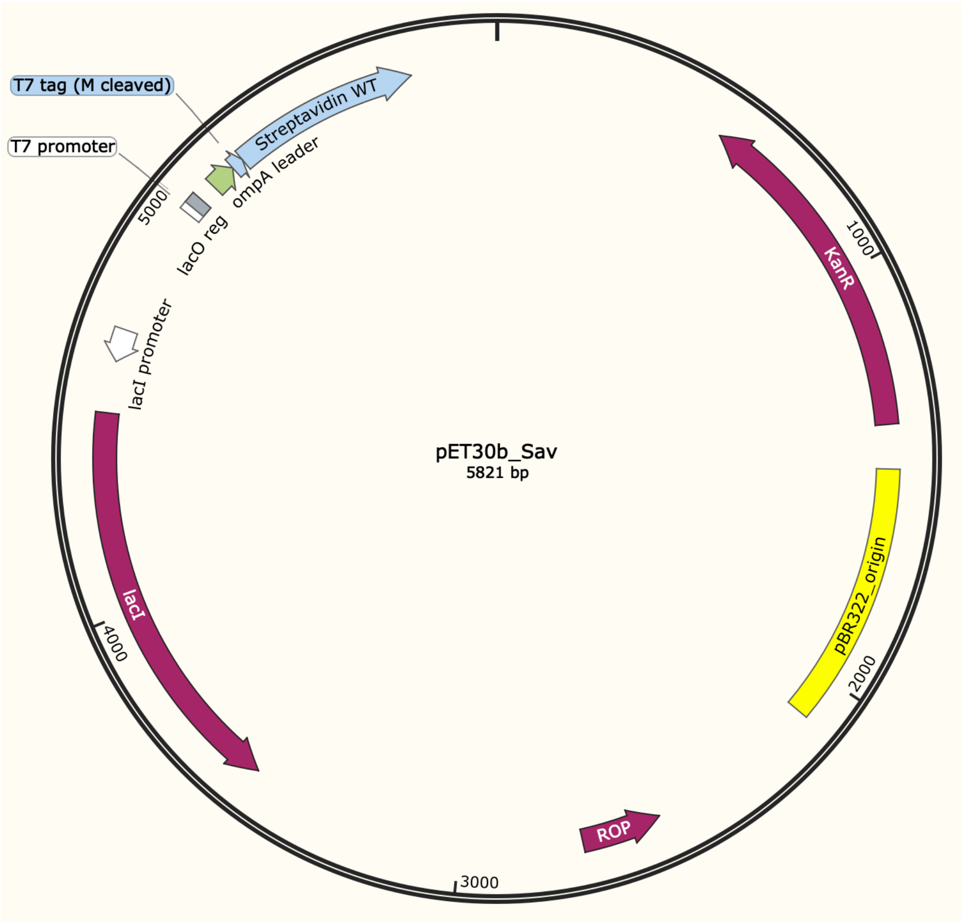

Supplementary Figure 4. Plasmid map of pET30b encoding Sav.

DNA sequence of periplasmic Sav. blue = positions S112 and K121.

ATGAAAAAGACAGCTATCGCGATTGCAGTGGCACTGGCTGGTTTCGCTACCGT
AGCGCAGGCCGCTAGCATGACTGGTGGACAGCAAATGGGTCGGGATCAGGCCGG
CATCACCGGCACCTGGTACAACCAGCTCGGCTCGACCTTCATCGTGACCGCGGGC
GCCGACGGCGCCCTGACCGGAACCTACGAGTCGGCCGTCGGCAACGCCGAGAGC
CGCTACGTCCTGACCGGTCGTTACGACAGCGCCCCGGCCACCGACGGCAGCGGC
ACCGCCCTCGGTTGGACGGTGGCCTGGAAGAATAACTACCGCAACGCCCACTCCG
CGACCACGTGGAGCGGCCAGTACGTCGGCGGCGCCGAGGCGAGGATCAACACCC
AGTGGCTGCTGACCTCCGGCACCACCGAGGCCAACGCCTGGAAGTCCACGCTGGT
CGGCCACGACACCTTCACCAAGGTGAAGCCGTCCGCCGCCTCCATCGACGCGGCG
AAGAAGGCCGGCGTCAACAACGGCAACCCGCTCGACGCCGTTCAGCAGTAATAA.

Protein sequence of periplasmic Sav. blue = positions S112 and K121.

MKKTAIAIAVALAGFATVAQAASMTGGQQMGRDQAGITGTWYNQLGSTFIVTAGA
DGALTGTYESAVGNAESRYVLTGRYDSAPATDGSGTALGWTVAWKNNYRNAHSATT
WSGQYVGGAEARINTQWLLTSGTTEANAWKSTLVGHDTFTKVKPSAASIDAAKKAGVN
NGNPLDAVQQ.

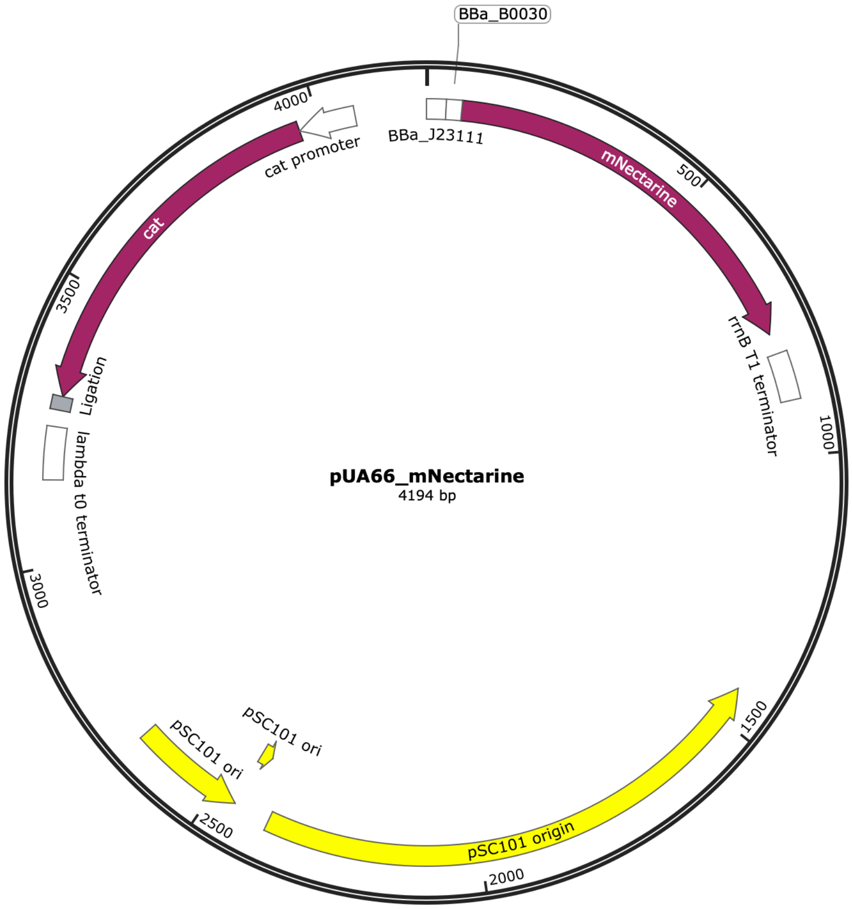

Supplementary Figure 5. Sequence of constitutively-expressed mNectarine. Plasmid map of pUA66 encoding mNectarine.

DNA sequence of mNectarine

ATGGTTAGCAAAGGCGAAGAAGATAACATGGCCATTATCAAGGAGTTTATGCGTTTCAAAGTTCATATGGAGGGTTCCGTCAACGGGCATGAGTTCGAGATTGAAGGCGAGGGGGAGGGCCGTCCGTACGAGGGTACACAAACAGCCAAACTGAAAGTCACGAAGGGTGGACCACTTCCGTTCGCGTGGGATATCCTGTCACCTCAATTCTGCTATGGAAGCAAAGCGTACGTGAAACATCCGGCCGATATTCCGGACTATCTGAAACTGTCGTTCCCTGAAGGTTTAAACTGGGAACGTGTGATGAACTTTGAGGACGGCGGGGTTGTTACCGTAACGCAGGATTCCTCTCTGCAAGACGGCGAGTTCATTTACAAGGTCAAACTCCGTGGTACAAATTTTCCCAGTGATGGTCCAGTTATGCAGTGTCGCACCGTTGGTTGGGAGGCCAGTACCGAACGTATGCATCCGGAGGACGGCGCACTCAAAGGCGAAATCATGCAACGCTTAAAGCTCAAGGACGGCGGTCATTACGACGCGGAAGTCAAAACAACTTACAAAGCAAAAAAACCTGTGCAGTTACCGGGTGCATATAACGTCGACATTAAACTCGATATTCTTTCCCATAACGAGGACTACACAATCGTTGAGCTGTACGAACGCGCGGAAGGCCGTCACAGCACGGGTGGGATGGACGAGCTCTATAAGTAATAA.

Protein sequence of mNectarine

MVSKGEEDNMAIIKEFMRFKVHMEGSVNGHEFEIEGEGEGRPYEGTQTAKLKVT
KGGPLPFAWDILSPQFCYGSKAYVKHPADIPDYLKLSFPEGLNWERVMNFEDGGVVTV
TQDSSLQDGEFIYKVKLRGTNFPSDGPVMQCRTVGWEASTERMHPEDGALKGEIMQR
LKLKDGGHYDAEVKTTYKAKKPVQLPGAYNVDIKLDILSHNEDYTIVELYERAEGRHSTG
GMDELYK.

Supplementary Table 1. Enrichment factor for the top 20 hits of the DE screening. The enrichment was computed as (count mutantX in top 5%)/(total count in top 5%)×(total count in DE)/(count mutantX in DE).

Mutant	DE count	Top 5% count	Enrichment in top 5%
FR	416	311845	741.8
MR	1072	384548	355.0
MW	575	71024	122.2
LQ	1201	51067	42.1
FQ	1642	28326	17.1
MI	1441	20822	14.3
YS	1022	13524	13.1
MY	834	9160	10.9
FN	325	3271	10.0
YA	1176	10761	9.1
FA	749	5136	6.8
FI	153	901	5.8
FS	601	3106	5.1
YL	1395	6892	4.9
DL	8849	40409	4.5
DQ	16938	76273	4.5
NR	24176	108007	4.4
YW	1037	4546	4.3
MA	2569	10807	4.2
MV	503	2093	4.1

Supplementary Table 2. Comparison of the top 20 mutants between 96-well plate experiment (activity measurements) and DE screening (enrichment). Overall, we found a 50% match in the top 10 hits and a 60% match in the top 20 hits.

rank	96-well plate screening	Activity (improvement over wt-Sav)	DE screening	Enrichment (over unsorted sample)
**1**	FQ	13.8	FR	741.8
**2**	FR	13.4	MR	355.0
**3**	MR	13.4	MW	122.2
**4**	MW	13.3	LQ	42.1
**5**	MI	13.0	FQ	17.1
**6**	AW	12.0	MI	14.3
**7**	FS	11.1	YS	13.1
**8**	MM	10.9	MY	10.9
**9**	LR	10.8	FN	10.0
**10**	FT	10.5	YA	9.1
**11**	MV	10.3	FA	6.8
**12**	FA	10.3	FI	5.8
**13**	FN	10.2	FS	5.1
**14**	FE	10.0	YL	4.9
**15**	YS	9.7	DL	4.5
**16**	IR	9.5	DQ	4.5
**17**	QR	9.4	NR	4.4
**18**	QI	9.2	YW	4.3
**19**	MA	9.1	MA	4.2
**20**	MY	9.1	MV	4.1

Supplementary Table 3. General reagents set-up for PCR.

**Reagents**	**Appropriate Amounts for one Reaction**
mQ H_2O	9.5 µL
2x Q5 maser mix	12.5 µL
Plasmid template (25 ng/µL)	1.00 µL
Forward Primer (10 µM)	1.00 µL
Reverse Primer (10 µM)	1.00 µL
Final volume for one reaction	25.0 µL

Supplementary Table 4. General PCR program for the library preparation.

**Cycle number**	**Denature**	**Anneal**	**Extend**
1	95 °C, 2 min		
2-25	95 °C, 15 s	65/72 °C, 20 s	72 °C, 2-5 min
27			72 °C, 10 min
28		4 °C	

Supplementary Table 5. Primers used for PCR amplification for Nanopore Sequencing/NGS.

Primer	Sequence
NGS_adapter_L1	aatgatacggcgaccaccgagatctacactctttccctacacgacgctcttccgatcttatcacgagaagcacgcattaataccc
NGS_adapter_L2	aatgatacggcgaccaccgagatctacactctttccctacacgacgctcttccgatctatcgatgtagaagcacgcattaataccc
NGS_adapter_L3	aatgatacggcgaccaccgagatctacactctttccctacacgacgctcttccgatctgatcttgtaagaagcacgcattaataccc
NGS_adapter_L4	aatgatacggcgaccaccgagatctacactctttccctacacgacgctcttccgatctcgatgccaatagaagcacgcattaataccc
NGS_adapter_L5	aatgatacggcgaccaccgagatctacactctttccctacacgacgctcttccgatcttcgatacagtgagaagcacgcattaataccc
NGS_adapter_R1	caagcagaagacggcatacgagatgtgactggagttcagacgtgtgctcttccgatcttacgttattcggacggcttcaccttg
NGS_adapter_R2	caagcagaagacggcatacgagatgtgactggagttcagacgtgtgctcttccgatctatttcaagaacggacggcttcaccttg
NGS_adapter_R3	caagcagaagacggcatacgagatgtgactggagttcagacgtgtgctcttccgatctgatgatctgcccggacggcttcaccttg
NGS_adapter_R4	caagcagaagacggcatacgagatgtgactggagttcagacgtgtgctcttccgatctcgattaacatagcggacggcttcaccttg
NGS_adapter_R5	caagcagaagacggcatacgagatgtgactggagttcagacgtgtgctcttccgatcttcgatgcgtcaaccggacggcttcaccttg

blue = i5 indices, violet = i7 indices, orange = barcodes, green = primer binding site.

References

[1] M. Jeschek, R. Reuter, T. Heinisch, C. Trindler, J. Klehr, S. Panke, T. R. Ward, *Nature* **2016**, *537*, 661–665.

[2] C. A. Woodall, in *E. Coli Plasmid Vectors: Methods and Applications* (Eds.: N. Casali, A. Preston), Humana Press, Totowa, NJ, **2003**, pp. 55–59.

[3] A. Zaslaver, A. Bren, M. Ronen, S. Itzkovitz, I. Kikoin, S. Shavit, W. Liebermeister, M. G. Surette, U. Alon, *Nat Methods* **2006**, *3*, DOI 10.1038/nmeth895.

[4] T. Völker, F. Dempwolff, P. L. Graumann, E. Meggers, *Angewandte Chemie - International Edition* **2014**, *53*, 10536–10540.

[5] G. Woronoff, A. el Harrak, E. Mayot, O. Schicke, O. J. Miller, P. Soumillion, A. D. Griffiths, M. Ryckelynck, *Anal Chem* **2011**, *83*, 2852–2857.

[6] T. Heinisch, F. Schwizer, B. Garabedian, E. Csibra, M. Jeschek, J. Vallapurackal, V. B. Pinheiro, P. Marlière, S. Panke, T. R. Ward, *Chem Sci* **2018**, *9*, 5383–5388.

[7] A. Stucki, P. Jusková, N. Nuti, S. Schmitt, P. S. Dittrich, *Small Methods* **2021**, *5*, 2100331.

[8] S. Deshpande, Y. Caspi, A. E. Meijering, C. Dekker, *Nat Commun* **2016**, *7*, 10447.
